# Working memory performance in euthymic bipolar patients

**DOI:** 10.1192/j.eurpsy.2022.918

**Published:** 2022-09-01

**Authors:** N. Charfi, A. Bouaziz, I. Gassara, R. Feki, N. Smaoui, S. Omri, M. Maalej, L. Zouari, J. Ben Thabet, M. Maalej

**Affiliations:** 1 Hedi Chaker University Hospital, Psychiatry, Sfax, Tunisia; 2 Hospital university of HEDI CHAKER, Psychiatry C Department, Sfax, Tunisia; 3 Hedi Chaker University Hospital, Psychiatry C, Sfax, Tunisia

**Keywords:** working memory, euthymic patients, bipolar disorder, Associated factors

## Abstract

**Introduction:**

Working memory (WM) deficit in bipolar disorder (BD) is heterogeneous and seems to be affected by both, demographic and clinical aspects, of the patient.

**Objectives:**

To assess the WM performance in euthymic bipolar patients (BP) comparing to healthy controls (HC) and to identify demographic and clinical factors associated with it.

**Methods:**

A case-control study was conducted among euthymic bipolar patients according to DSM-5. The recruitment of patients was performed in the outpatient psychiatric unit in the university hospital Hedi Chaker in Sfax during the period from January to December 2020. The HC were matched to BP on gender, age and education level. The Screening for Cognitive Impairment in Psychiatry scale (SCIP) was used to assess the WM performance by the WM test (WMT).

**Results:**

We recruited 61 BP (37 males and 24 females) and 40 HC (20 males and 20 females). The average age of BP was 41.75 years (SD=11.6 years). The BD group included 47 BD type I and 14 BD type II patients. The mean duration of illness was 9.75 years (SD=7.93 years). Thirty-seven BP (60.7%) had a history of psychotic symptoms. The WMT score was significantly lower among BP than HC (p<0,001). The female gender, the type II of BD and the history of psychotic symptoms correlated with a poorer performance on WMT (p=0.019; 0.017 and 0.002, respectively).

**Conclusions:**

BP have shown significant impaired performance in WM even during euthymia. Female gender of patient, type II of BD and psychotic symptoms seem to be the predictors of this impairment.

**Disclosure:**

No significant relationships.

